# Design of Potent and Controllable Anticoagulants Using DNA Aptamers and Nanostructures

**DOI:** 10.3390/molecules21020202

**Published:** 2016-02-06

**Authors:** Abhijit Rangnekar, Jessica A. Nash, Bethany Goodfred, Yaroslava G. Yingling, Thomas H. LaBean

**Affiliations:** 1 Department of Materials Science and Engineering, College of Engineering, North Carolina State University, Raleigh, NC 27695, USA; abhi1jit@gmail.com (A.R.); janash@ncsu.edu (J.A.N.); yara_yingling@ncsu.edu (Y.G.Y.); 2 Department of Molecular and Structural Biochemistry, College of Agriculture and Life Sciences, North Carolina State University, Raleigh, NC 27695, USA; bgoodfred@gmail.com

**Keywords:** thrombin, DNA aptamers, anticoagulation, DNA nanotechnology

## Abstract

The regulation of thrombin activity offers an opportunity to regulate blood clotting because of the central role played by this molecule in the coagulation cascade. Thrombin-binding DNA aptamers have been used to inhibit thrombin activity. In the past, to address the low efficacy reported for these aptamers during clinical trials, multiple aptamers have been linked using DNA nanostructures. Here, we modify that strategy by linking multiple copies of various thrombin-binding aptamers using DNA weave tiles. The resulting constructs have very high anticoagulant activity in functional assays owing to their improved cooperative binding affinity to thrombin due to optimized spacing, orientation, and the high local concentration of aptamers. We also report the results of molecular dynamics simulations to gain insight into the solution conformations of the tiles. Moreover, by using DNA strand displacement, we were able to turn the coagulation cascade off and on as desired, thereby enabling significantly better control over blood coagulation.

## 1. Introduction

**Figure 1:**
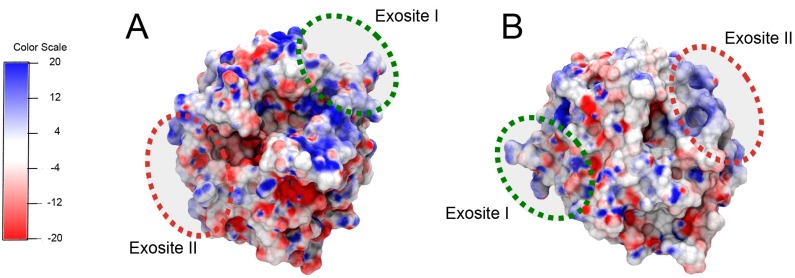
Thrombin structure (pdbID: 1ppb) [4]. (**A**,**B**) present two views of the protein. The binding sites of the aptamers (exosites I and II) are highlighted with green and red circles, respectively. Thrombin’s surface is colored by electrostatic potential, where red corresponds to negative potential and blue corresponds to a positive potential (units are kT/e) [5,6,7].

Thrombin is a crucial component of the blood coagulation cascade. It catalyzes key reactions during coagulation, including conversion of Factor XI to XIa, Factor VIII to VIIIa, Factor V to Va and soluble fibrinogen to insoluble fibrin. Thrombin also causes activation and aggregation of platelets during blood clot formation [1]. Regulation of thrombin activity via inhibition can play a crucial role in clinical conditions such as acute coronary syndrome, peripheral vascular disease and deep vein thrombosis, as well as arterial and venous thromboembolism [2]. Thrombin activity can be modulated by blocking the substrate-binding exosites I and II (Figure 1). The anion-binding exosite I helps in conversion of fibrinogen to fibrin and the heparin-binding exosite II plays a crucial role in platelet activation and aggregation during coagulation [1]. Several direct thrombin inhibitors, such as hirudin, bivalirudin, argatroban, dabigatran, *etc.* are currently available for clinical use. However, many of them have either severe side-effects or they suffer from narrow therapeutic windows [3]. Nucleic acid aptamers are non-toxic and non-immunogenic and thus offer a safer alternative [2].

Dozens of aptamers have been developed over the last two decades, several of which are being developed as therapeutics and are in various phases of clinical trials for the treatment of disorders including acute myeloid leukemia, non-small-cell lung cancer, vein graft failure, *etc.* [8]. Aptamers targeting von Willebrand Factor and thrombin are also undergoing clinical trials having potential applications in coagulation-related disorders [8,9]. An FIXa targeting aptamer is also currently being developed as an intravenous anticoagulant for clinical use during surgery [10]. Another important advantage of nucleic acid aptamers is that their activity can be modulated using a specific antidote—a single-stranded DNA (ssDNA) oligonucleotide with a sequence complementary to the aptamer sequence. The thermodynamically more stable Watson-Crick base-pairing between the aptamer and antidote disrupts the less stable tertiary structure of the aptamer and thereby, its function [11].

**Figure 2:**
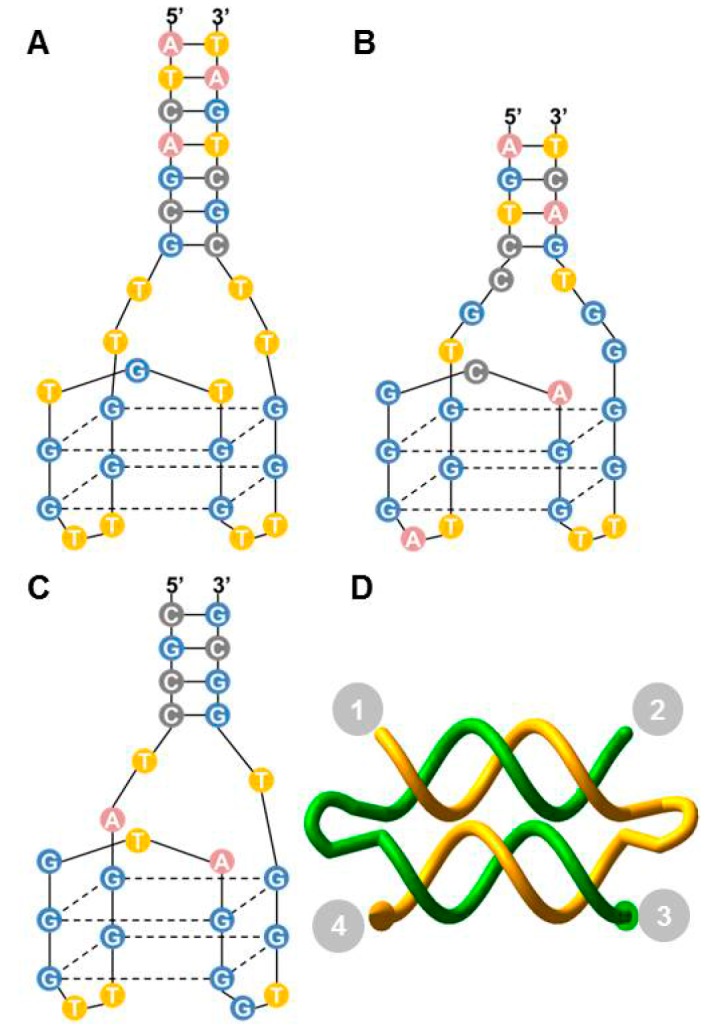
Thrombin-binding aptamers and DNA weave tile. (**A**) Apt A (also named HD1) with a 7-bp stem and -TT- linkers at the ends; (**B**) Apt B (also named HD22); (**C**) Apt P (also called NU172). The structure shown is a model due to the absence of a published crystal structure; (**D**) A two-helix weave tile (2HT) with the four ends numbered for aptamer positioning and assembly naming.

Several aptamers specifically target the two exosites of thrombin including: Aptamer HD1 (hereafter called Apt A) binds to exosite I (Figure 2A) and aptamer HD22 (hereafter called Apt B) binds to exosite II (Figure 2B). Apt A has significant anticoagulant activity in clinical clotting assays [3,12]. It also binds to prothrombin with high affinity resulting in inhibition of prothrombin activation. Apt B binds to exosite II with very high affinity [3], but it is not a good thrombin inhibitor in platelet-deficient plasma clotting assays. Aptamer NU172 (hereafter referred to as Apt P) also binds to exosite I (Figure 2C), but with a much higher affinity compared to Apt A [8]. It is currently undergoing clinical trials. In addition to the thrombin-binding DNA aptamers, an RNA aptamer called TOG25 that targets exosite II, has also been reported [9].

Apt A was approved for clinical trials as an anticoagulant during bypass surgery. However, it could not be developed further due to suboptimal dosing profile [9]. Subsequently, attempts have been made to improve the efficacy of Apt A. Studies have shown that creating multivalent constructs by concatenating 2–4 copies of the aptamer significantly increases its activity [13,14,15]. Design of multivalent aptamers may provide a protective mechanism against degradation by nucleases and may also decrease renal clearance, resulting in enhanced circulation time. Incorporation of multiple aptamers in a pre-programmed manner may also improve the overall binding affinity due to synergy between the binding domains. Another reason for the improved performance of multi-aptamer molecules may be increased local concentration, meaning that even if an aptamer dissociates from its binding site, being tethered to other bound aptamers will limit its ability to diffuse away and thereby increase its probability of rebinding. We have shown that four copies of Apt A, when displayed on a DNA weave tile nanostructure, showed much higher anticoagulant activity than individual Apt A owing to the synergistic effects and higher local concentration [16].

A second strategy for improving thrombin inhibition involves linking Apt A and Apt B using an ssDNA linker [3]. However, since inter-aptamer distance and relative orientation appear crucial for optimal binding, DNA nanostructures provide a more ideal platform for multi-ligand presentation. We have demonstrated the use of a DNA weave tile (Figure 2D) as a platform for linking aptamers with significant control over spacing and relative orientation [17]. Moreover, the moderate flexibility of weave tiles improves the overall binding of the construct to thrombin by enabling it to conform more closely to positively charged regions on the thrombin surface (Figure 1). We have previously demonstrated the complete and rapid reversal of anticoagulant activity by targeting Apt A on weave tile using ssDNA antidote [17]. Reversal of Apt A activity was more stable for aptamer displayed on weave tile compared to free aptamer. We further demonstrated that thrombin-binding aptamers devoid of chemical modifications are much more stable in plasma when linked to weave tile compared to free aptamers [17].

The detailed mechanism behind the increased efficacy on weave tiles compared to free aptamers or ssDNA-linked aptamers is not yet well understood. Molecular dynamics simulations of biomolecules have long been used to study phenomena such as protein folding or conformational transitions of DNA [18,19]. Recently, atomistic molecular dynamics have been applied to larger DNA nanostructures such as tiles or small origami [20,21]. To gain a better understanding of the flexibility and conformational space explored by weave tiles (which may aid interactions with thrombin) we have employed here all-atom molecular dynamics simulations of several weave tile structures. Our previous studies [17] have shown an effect from both the number of helices on the weave tile and the distance between aptamer connection points. We expect results from these simulations to provide insights into differences in tile flexibility for different helical linking geometries; these insights will then be used to correlate and understand changes in anticoagulant function.

One disadvantage of weave tile anticoagulants is the relatively high cost of chemical synthesis of oligonucleotides. In order to overcome this obstacle, we have switched from Apt A to Apt P, since Apt P has much higher binding affinity for thrombin and, in conjunction with Apt B, is expected to inhibit thrombin more potently. Consequently, lower concentrations of construct should be needed to achieve desired levels of anticoagulation, thereby reducing cost. As stated earlier, use of antidotes provides significant control over anticoagulation. We further demonstrate below that by using fundamental properties of DNA such as strand-displacement, it is possible to effectively build an anticoagulation system with an even higher degree of control where the coagulation cascade can be turned on and off repeatedly, as desired.

## 2. Results

### 2.1. Comparison of Anticoagulant Activity of the Aptamers

**Figure 3:**
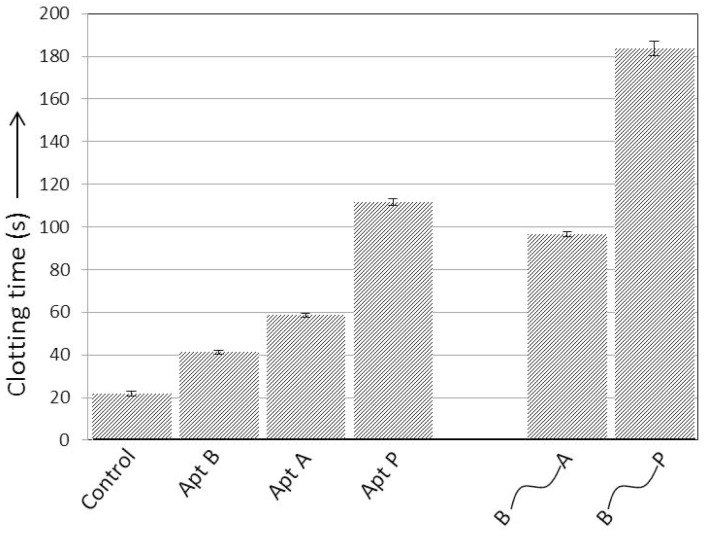
Anticoagulant activity of thrombin-binding aptamers. Apt P demonstrates much better anticoagulant activity than Apt A. Anticoagulant activity improves when the aptamers are linked via a 20-base ssDNA linker, with the B-P pair showing much better anticoagulation than the B-A pair. The concentration of construct in each case is 1 µM. Clotting time is expressed as mean ± SEM.

Activated partial thromboplastin time coagulation assays (aPTT) were performed to determine the anticoagulant potential of Apt A, Apt B and Apt P (Figure 3). As expected, exosite II-binding Apt B did not show significant anticoagulant activity on its own due to the use of platelet deficient plasma. Moreover, higher potency of Apt P as compared to Apt-A was confirmed. Additionally, when the aptamer pairs B-A and B-P were connected via a 20-nucleotide ssDNA linker, the resulting construct displayed much higher anticoagulant activity compared to free aptamers (Figure 3) which substantiates previously published results [3,17].

### 2.2. Molecular Dynamics—Structural Analysis of Weave Tiles (without Aptamers)

**Figure 4:**
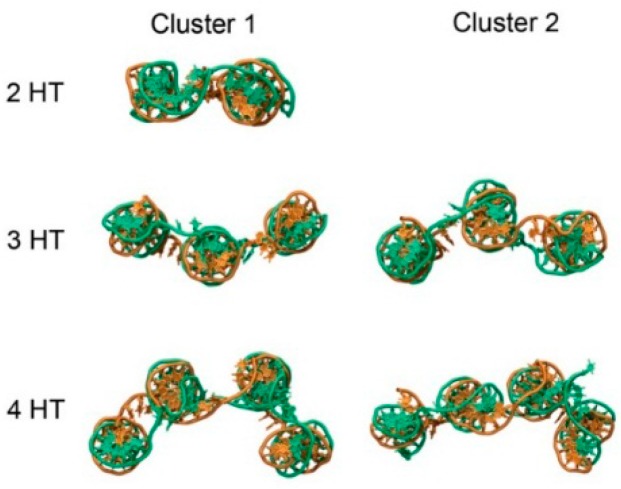
Snapshots showing representative structures of the first two conformational clusters from MD trajectories of weave tiles. According to the clustering metric used, 2HT had a total of 1 cluster, the 3HT had 5 and the 4HT had 7. The first cluster (Cluster 1) is the most common conformation of the tile, while Cluster 2 is the second most common conformation observed during simulations.

In order to examine weave tile structural flexibility and the conformational ensembles sampled, three sizes of weave tiles, without appended aptamers, were simulated using all-atom molecular dynamics (MD). Analysis was performed on the last 120 ns of simulation time (for simulations with total times of at least 180 ns) using CPPTRAJ [22], part of the AmberTools suite and Curves+ [23], a program for analyzing nucleic acid conformations. Clustering analysis for molecular dynamics simulations groups similar conformations and provides insight into the range of the conformational space visited by the molecule. Tile conformations were grouped using a hierarchical agglomerative cluster algorithm [24] based on RMSD of the DNA backbone. A critical distance of 15 Å for the RMSD was used to determine conformational clusters (Figure 4). It was shown that larger tiles (with more connected helices) provided larger numbers of distinct conformational clusters, indicating greater conformational fluctuation of the structure.

### 2.3. Weave Tile-Based Anticoagulation

**Figure 5:**
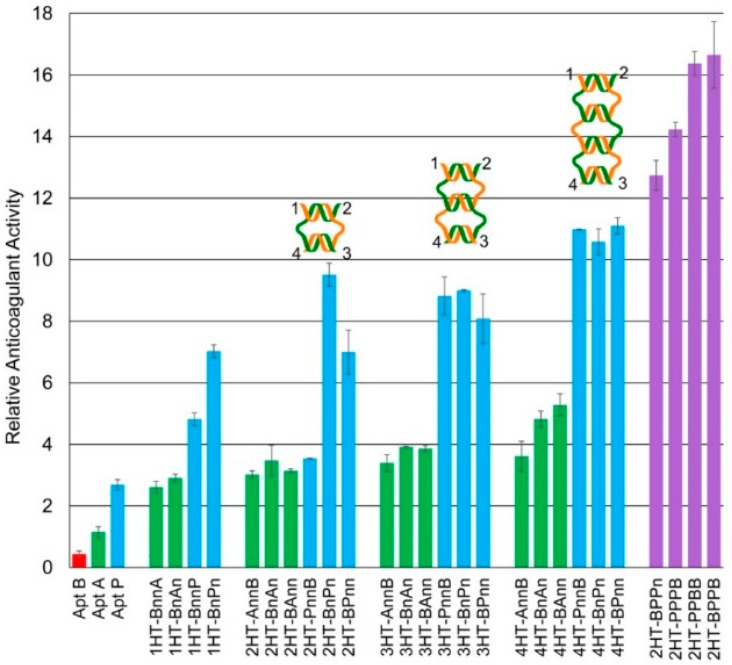
Relative anticoagulant activity of weave tile constructs. Weave tiles with B-P aptamer pairs (blue) demonstrate much better anticoagulant activity compared to those with B-A pairs (green). Activity increases with tile size, except for 2HT-BnPn which shows better activity than 3HT-BnPn. Incorporating multiple copies of Apt P on 2HT-BnPn improves anticoagulation due to higher local concentration (purple). However, incorporating multiple copies of both Apt P and B yields the most effective anticoagulants in 2HT-PPBB and 2HT-BPPB. Weave tile construct names bear a 4-tuple that provides the aptamer identity or “n” for no aptamer for each of the four numbered strand ends (a more thorough explanation of this nomenclature is provided in Section 4.2). Relative anticoagulant activity is calculated as described in Materials and Methods. Error bars represent SEM. Raw clotting times are provided in the Supplementary Materials (Table S2). Relative anticoagulant activity of free aptamer samples is shown for Apt B (red), Apt A (green) and Apt P (blue).

Coagulation assays of weave tiles bearing aptamer pairs B-A and B-P were performed (Figure 5). Weave tiles ranging from the one-helix tile (1HT) to the four-helix tile (4HT) were used and the aptamer pairs were positioned in different configurations, as illustrated in Figure 5. For naming the various configurations, available positions are numbered as shown in Figure 2D and we adopted tile names, such as 2HT-1234. Here, depending on the position of the aptamers, numerals are replaced by A, B or P (depending upon the aptamer used at each position), or n (when there is no aptamer). While a gradual increase in clotting time was observed from 1HT to 4HT for B-A and B-P aptamer pairs, the clotting times for the B-P pair were significantly higher compared to B-A pair. However, since 2HT-BnPn yielded a longer clotting time than 3HT and approaching that of 4HT, we used 2HT for the next round of experiments. In the first instance, two and three copies of Apt P were displayed on 2HT (BPPn and PPPB). As expected, they demonstrated higher clotting time than 2HT-BnPn containing a single copy of Apt P. Furthermore, when two copies each of the Apt B and P were used on 2HT (PPBB and BPPB), it resulted in even better anticoagulation with relative anticoagulant activity of more than 16 compared to approximately 11 for 4HT (Figure 5).

### 2.4. Antidote Studies

**Figure 6:**
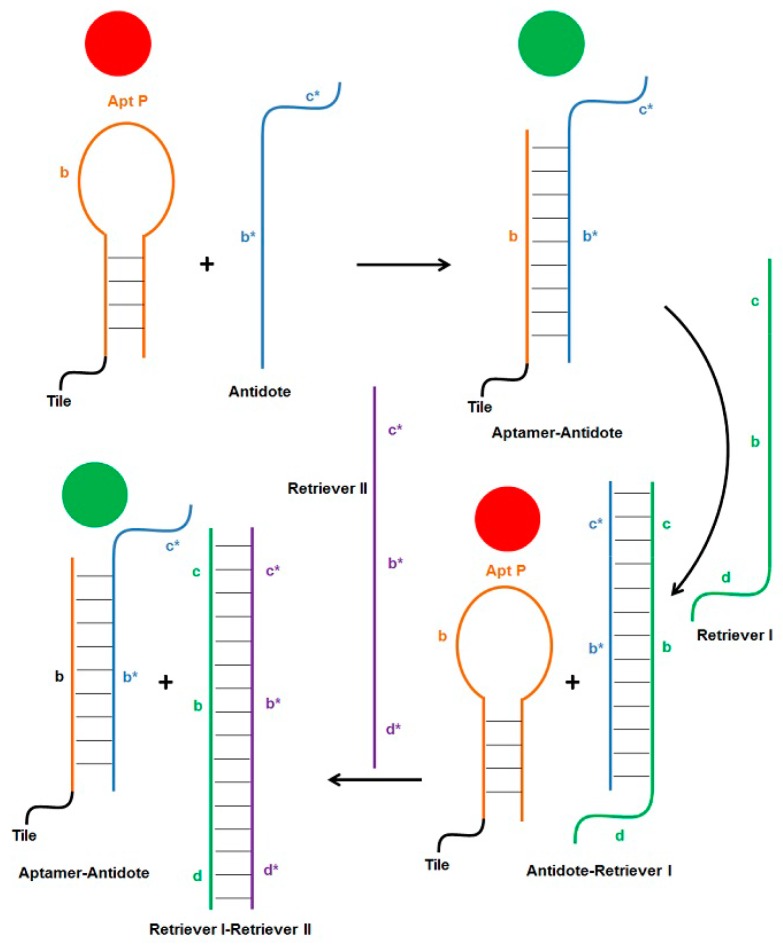
Schematic of regulation cycles. Aptamer turns off the coagulation cascade (red circles), while antidote reverses inhibition and turns on coagulation (green circles). Coagulation can be turned off again by addition of retriever I strand which binds to the overhang in the antidote and displaces the aptamer, which regains its folded structure and inhibits thrombin. Similarly, retriever II strand can displace antidote using the overhang on retriever I. The now free antidote, once again, binds to and deactivates aptamer, turning coagulation back on.

**Figure 7:**
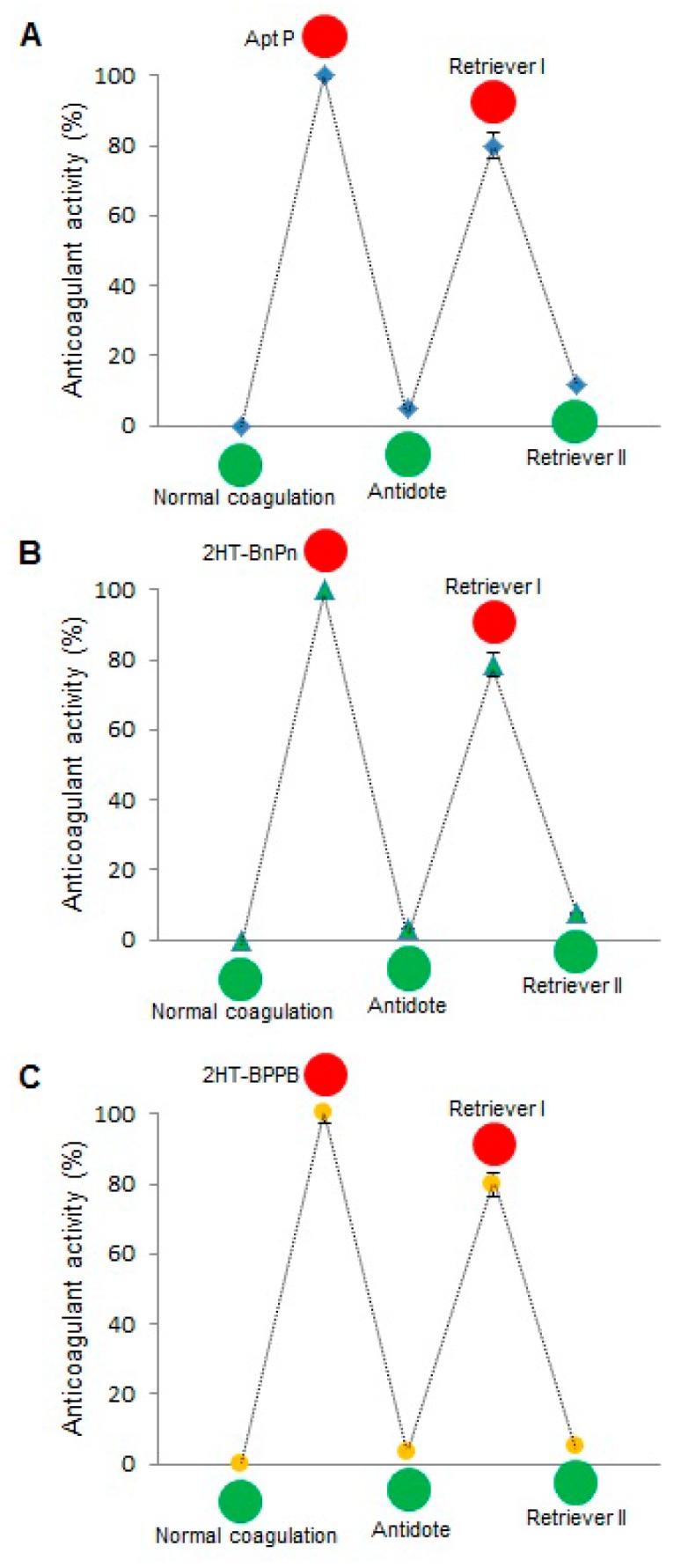
Cycles of antidote/retriever regulation in aPTT assays using (**A**) Apt P; (**B**) 2HT-BnPn and (**C**) 2HT-BPPB. Addition of antidote reverses anticoagulation to less than 10%, whereas addition of retriever I restores it to almost 80%. Retriever II reverses anticoagulation back to less than 10%. The % anticoagulant activity is calculated as described in Materials and Methods. Error bars represent SEM. Raw clotting times are provided in the Supplementary Materials (Table S4).

After establishing 2HT-PPBB and 2HT-BPPB as the most effective anticoagulant constructs, the next step was to determine the extent of control which can be exercised over these inhibitors. Toward this end, an antidote and two retriever strands were used as explained in Figure 6. Addition of antidote unfolds and deactivates aptamer and reverses anticoagulation. Retriever I binds antidote and reactivates aptamer, thereby turning off coagulation. Similarly, retriever II frees the antidote which then binds aptamer and turns on coagulation. This regulation system was tested for free Apt P, and for constructs 2HT-BnPn and 2HT-BPPB. As expected, addition of antidote and retriever II reversed the anticoagulation to around 10% or less, whereas retriever I restored it to almost 80% in all three cases (Figure 7).

## 3. Discussion

The activated partial thromboplastin time (aPTT) assay is among the most commonly used techniques in clinical and research settings to study blood clotting [11]. In this study, aPTT was used to characterize the clotting of platelet-deficient plasma. Two previously reported results were confirmed: Apt B, which binds exosite II, had minimal effect on clotting time; and Apt P is a significantly better anticoagulant than Apt A (Figure 3), as expected due to its higher affinity for exosite I [8]. Previous studies have shown that linking Apt B and Apt A via ssDNA for simultaneous targeting of the two exosites improves the anticoagulant activity over individual aptamers [3,17]. Here, similar results were obtained when the two aptamers were connected using a 20 nucleotide ssDNA linker. Moreover, when Aptamers B and P were connected in similar fashion, much better anticoagulant activity was observed (Figure 3).

It has also been shown previously that using DNA weave tiles as linking platform for Aptamers B and A further improves their activity over ssDNA linker [17]. Thus, it was logical to use weave tiles for B-P aptamer pairs to obtain even more potent anticoagulants than previously designed. In addition to providing an optimum degree of flexibility, weave tiles also offer control over inter-aptamer spacing and orientation. Moreover, the weave tile can conform to the thrombin surface and improve the overall affinity for thrombin by interacting with the positively charged thrombin surface between the two exosites, as illustrated in Figure 1, thereby aiding in anticoagulation.

Efficiency of the tile as a scaffold is expected to depend on distance between the binding sites of aptamers, as well as the flexibility of the tile. In order to better understand the likely interactions between weave tile and thrombin, molecular dynamics simulations were performed with 2HT, 3HT and 4HT. The ends of the weave tiles were labeled as 1, 2, 3, and 4, as illustrated in Figure 2D. Analysis of end-to-end distance between attachment points 1-2, 1-3, and 1-4 in simulations is shown in Table 1. The error represents standard deviation and signifies the extent of the movement (degree of flexibility) experienced by aptamers connected at the specified corners. Higher standard deviation indicates greater fluctuation of the value. It can be seen that 1-3 configuration is the most flexible for each of the tile sizes while 1-2 configuration is the least flexible. These results are along expected lines, since double-stranded DNA of this size (16 bp) is known to be almost linear with fairly low flexibility [25] and the largest conformational fluctuations should be between neighboring helices.

**Table 1:** Distance in angstroms between connection points on each tile observed in molecular dynamics simulation. Uncertainty associated with each measurement is represented by the standard deviation of the data. 1-to-2 represents the distance along one helix, 1-to-3 is the diagonal across a tile, and 1-to-4 is the distance between helices on one tile edge.

Tile	1-to-2	1-to-3	1-to-4
2HT	47.6 ± 1.8	55.2 ± 3.4	21.4 ± 3
3HT	49.1 ± 1.8	79.3 ± 5.9	62.3 ± 5.1
4HT	49.3 ± 1.6	84.9 ± 6.7	83.1 ± 6.6

Trajectories were grouped into clusters using a hierarchical agglomerative method, using the root-mean-square deviation of the DNA backbone as a metric. According to this metric, the number of clusters were 1, 5 and 7 for the 2HT, 3HT and 4HT, respectively. A higher number of clusters indicates greater conformational space explored by the tile. Shown in Figure 4 are snapshots of representative structures for each tile. Clusters with a lower number represent conformations observed more often in the ensemble. For the 2HT, the specified criteria led to only one cluster. Representative structures from clustering show that movement of the tiles occurred primarily through movement of the helices relative to one another (Figure 4). The 1-3 configuration derives its flexibility from the axial flexibility of the helices as well as the flexibility of the -TTTT- linkers connecting the helices, making it the most flexible configuration. Similarly, 1-4 configuration displays intermediate flexibility, owing mainly to the -TTTT- linkers. Not surprisingly, the flexibility of 1-3 and 1-4 configurations increases with the size of the tile, since addition of each helix introduces a new axis and, thereby, a new degree of freedom.

The use of -TTTT- linkers imparts conformational freedom to the tiles. Because the 2HT has only two helices, the tile adopts a predominantly planar conformation, and only one cluster is observed (Figure 4). The 3HT and 4HT, however, show conformations with helices able to move relative to one another. A third helix on the tile allows for bending with the center helix as a hinge (Figure 4). To better quantify the range of motion experienced by the weave tile, we measured the angle formed by the three helices. Two planes may be defined on the 3HT using the end base pairs on the outer helix and the center of the middle helix. The angle between the defined planes for clusters 1 and 2 of the 3HT was 134° and 218°, respectively. The tile experiences angles throughout this range, indicating a high degree of flexibility. The four helix tile is able to explore an even greater range of conformational space since all four helices can move relative to one another.

In aPTT assays on weave tiles, ranging from 1HT to 4HT, it was found that B-P aptamer pairs gave much higher anticoagulant activity than B-A pairs for the same tile and configuration (Figure 5). This can easily be attributed to the higher thrombin inhibition activity of Apt P over Apt A. Moreover, 1-3 configuration for both sets of aptamers (BnAn and BnPn) performed better than other configurations on the same tile. These findings, in conjunction with molecular dynamics results, indicate that flexible configurations provide a boost in thrombin inhibition activity. It should be noted here that 1-3 configuration is not the best for 4HT for either set of aptamers (4HT-BnAn and 4HT-BnPn). This may be because 4HT is large and has more points of interactions with thrombin, possibly increasing affinity of the complex, and thus requiring less flexibility. In addition to flexibility, anticoagulant activity depends on inter-aptamer spacing and relative orientation. A construct with optimal inter-aptamer spacing and orientation for targeting both exosites may not require as much flexibility as another structure which does not have optimum spacing or orientation. In the final analysis, all three factors—flexibility, inter-aptamer spacing and relative orientation—play crucial roles.

Weave tiles of different sizes and configurations provide different inter-aptamer spacing and orientation, they will conform differently to the thrombin surface, and, therefore, require different degrees of flexibility to target both exosites simultaneously. Weave tiles increase the on-time of thrombin-aptamer complex due to higher overall affinity of the construct to thrombin. The on-time in a protein-ligand interaction is characterized as the amount of time the ligand is bound to the protein before dissociating. Thus, greater affinity between protein and ligand leads to higher on-time, and thus better inhibition. Similarly, the off-time is characterized as the amount of time the ligand is free in solution and not bound to the protein. Off-time should be related to the size and concentration of the ligand. The higher on-time of the tile/aptamer constructs compensates for the possibly higher off-time of the fairly bulky weave tile structures. The combination of all these factors, thus, results in higher affinity between thrombin and aptamers on weave tile as compared to free aptamers, which gives higher anti-thrombin activity and better anticoagulation.

It may be noted from Figure 5 that the anticoagulant activity increases with the size of the tile, except for 2HT-BnPn. This construct has higher activity compared to other 2HT configurations as well as to all the 3HT configurations. The same is, however, not true for 2HT-BnAn. This illustrates that while designing an effective inhibitor, the size of the weave tile and configuration of aptamers for optimum inhibition depend on which aptamers are used. 2HT-BnPn has slightly lower anticoagulant activity than 4HT constructs, but due to its smaller size, it is much less expensive to synthesize. Considering the lower cost of synthesis and its significantly higher anticoagulant activity compared to similarly sized constructs, 2HT-BnPn was used as a model anticoagulant for further studies.

It has been demonstrated that increasing the local concentration of aptamers increases their anticoagulation activity [16,17]. Here, 2HT-BnPn was labeled with additional copies of Apt P to further increase its local concentration while maintaining the concentration of tile complex at 1 µM. Anticoagulant activity of the resulting constructs (2HT-BPPn and 2HT-PPPB) is shown in Figure 5. As expected, two copies of Apt P on the weave tile (2HT-BPPn) was significantly better than 2HT-BnPn, and three copies of Apt-P (2HT-PPPB) was the best owing to the highest local concentration of Apt P. Moreover, having multiple copies of Apt P on a smaller tile (2HT) was more effective than having a single copy on larger tiles (4HT). In this design, however, there was only one copy of Apt B targeting exosite II, but multiple copies of Apt P targeting exosite I. Although having more copies of Apt P caused the observed increase in the anticoagulant activity in Figure 5, it was hypothesized that having more than one copy of both the aptamers targeting the two exosites should significantly increase the overall binding affinity of the construct to thrombin, and therefore the on-time of the weave tile on thrombin, resulting in even higher clotting times. This hypothesis was confirmed when two copies each of Apt P and Apt B were incorporated into 2HT in two different configurations (2HT-PPBB and 2HT-BPPB), and these constructs showed the best anticoagulant activity observed so far. Activity of these constructs was more than 16 times better than the free Apt A, and almost seven times more potent than free Apt P. Thus, by suitable choice of weave tile and aptamers as well as placement of multiple copies of those aptamers on the tile, we were able to obtain highly potent anticoagulants which are significantly more effective and considerably less expensive due to their smaller size than the ones previously obtained [17].

Reversal of the anticoagulant activity of thrombin-binding aptamers using single-stranded RNA or DNA antidote has been demonstrated previously [11,17,26]. We have also shown that this reversal is more stable when the aptamer is on weave tile [17]. Single-stranded antidote, complementary to aptamer sequence, forms stable Watson-Crick pairing with the aptamer bases, thereby disrupting the less stable G-quadruplex structure of the thrombin-binding aptamer, which results in loss of anticoagulant activity. The use of short, non-toxic, and non-immunogenic DNA strands to control the coagulation cascade offers many advantages over currently used compounds. The principle of strand displacement can be employed further to dissociate the aptamer-antidote complex in order to restore the functionality of the aptamer. As illustrated in Figure 6, addition of Apt P (free or attached to the weave tile) turns the coagulation cascade off (red circle), which can be turned back on (green circle) by addition of the antidote strand. Sequence b* of the antidote forms Watson-Crick pairing with aptamer sequence b. Additionally, an overhang sequence c* was added to the antidote such that another strand (called retriever I) which includes sequence b-c can be used to first bind overhang c in the antidote. This will displace aptamer from complex by forming the more stable antidote-retriever I complex, thereby making the aptamer functional again and turning the coagulation cascade off (Figure 6). The retriever I strand also has an overhang sequence d, which can then be exploited by retriever II strand to displace antidote from the complex. Now free antidote can again bind to aptamer and inactive it, turning coagulation on again. This hypothesis was first tested and confirmed with 2HT-BnPn using non-denaturing PAGE, the results of which are shown in Supplementary Figure S2. When this scheme was tested by aPTT assay, the results were very encouraging (Figure 7). Free Apt P, dual aptamers on weave tile (2HT-BnPn) and two copies of each aptamer on weave tile (2HT-BPPB) were tested. As expected, addition of antidote brought the anticoagulant activity in each case to less than 10%, which was restored to more than 80% after addition of retriever I. Addition of retriever II reduced the anticoagulant activity again to less than 10%, thus demonstrating rapid regulation of anticoagulant activity.

## 4. Experimental Section

### 4.1. DNA Strands and Weave Tile Formation

Synthetic oligonucleotides were purchased from Integrated DNA Technologies (Coralville, IA, USA) in unpurified form and used as such. Sequences of all DNA strands used in this study are provided in the Supplementary Material. Aptamers and weave tiles were folded by annealing stoichiometric mixtures of the participating strands (10 µM) in a buffer consisting of 20 mM HEPES (pH 7.4), 150 mM NaCl and 2 mM CaCl_2_. Annealing was performed by incubating samples at 90 °C for 5 min, followed by room temperature incubation for 5 min and incubation on ice for 5 min (see Supplementary Figure S1 for PAGE characterization of the weave tiles). Samples were subsequently kept at room temperature for immediate use.

### 4.2. Weave Tile Structures and Nomenclature

The weave tiles consist of 16-bp long helical domains, connected on the ends by -TTTT- loops. A weave tile constructed with two helical domains is thus called 2HT (Figure 2D). Aptamers can be appended to the ends of the oligonucleotide strands of the weave tile, decorating the four corners of the tile. For naming the various aptamer configurations on the weave tile, available positions are numbered as shown in Figure 2D and we adopted tile names such as: 2HT-XXXX. Based on the positioning of the aptamers, X would be replaced by A, B or P (when the tile has Apt A, Apt B or Apt P at that position), or n (when there is none). Apt A is connected to the tiles via a 7-bp stem and a -TT- spacer [16].

### 4.3. Computational Studies of Weave Tile (without Appended Aptamers)

#### 4.3.1. Weave Tile Models

DNA tiles were constructed with helices parallel and coplanar with 25 angstrom distance between their respective centers of mass as a first approximation for the simulations [27]. Helices were then connected with -TTTT- loops to form the completed tile.

#### 4.3.2. Molecular Dynamics Simulations

Structures were solvated in a truncated octahedral box with a 12.0 angstrom buffer of TIP3P water with explicit sodium counterions for charge neutralization. After structures were neutralized, additional salt (NaCl) was added to a concentration of 0.1 M. The ff12SB force field in Amber was used with ion parameters by Joung and Cheatham [28] for all simulations. Structures were subjected to 10,000 steps of steepest descent minimization, followed by 100 picoseconds of heating to 300 K in the isothermal-isobaric (NPT) ensemble with Langevin thermostat to allow equilibration of solvent before production simulations. Production simulations were run with Amber12 using the pmemd.cuda accelerated GPU code [29,30,31] in the NPT ensemble. Each tile was simulated for at least 180 nanoseconds, of which the last 120 ns was used for analysis.

### 4.4. Coagulation Assay

A model ST4 coagulometer (Diagnostica Stago, Parsippany, NJ, USA) was used to run activated partial thromboplastin time (aPTT) assays to determine the clotting time for each sample. Pooled human plasma was purchased from George King Bio-Medical (Overland Park, KS, USA). CaCl_2_ solution and TriniClot aPTT reagent were purchased from Trinity Biotech USA (Jamestown, NY, USA). 50 μL plasma was activated with 50 μL TriniClot aPTT reagent and incubated at 37 °C for 5 min. Subsequently, 16.67 μL of control buffer or 10 µM sample was added followed by further incubation for 5 min at 37 °C. Clotting was initiated by adding 50 μL CaCl_2_ solution, and all tests presented here were conducted in triplicate at the same final construct concentration (1 μM). The coagulometer measured the time for clot formation after the addition of CaCl_2_; clotting times were recorded and analyzed. All data presented here is the average of tests conducted on at least two different days. Data for each sample is represented either as the average of all the clotting times for the sample obtained over all the conducted tests or as the relative anticoagulant activity (described below). Error bars represent the standard error of the mean (SEM).

### 4.5. Switching On-Off the Coagulation Cascade

Plasma was activated and treated with aptamer as described above. Antidote was subsequently added to achieve an antidote:aptamer ratio of 2:1, then the reaction was incubated for 5 min at 37 °C. Retriever I and retriever II were subsequently added (at concentrations equal to antidote) with 5 min of incubation at 37 °C after each step, followed by CaCl_2_ activation. Volumes and concentrations of aptamer/antidote/retriever strands were appropriately adjusted for final concentrations of 1 µM aptamer construct.

### 4.6. Anticoagulant Activity Calculation

The relative anticoagulant activity is calculated as:

(1) (T_sample_ − T_control_)/T_control_

where T is the clotting time in seconds, so a relative anticoagulant activity of 1 is equivalent to twice the clotting time of the buffer control.

Normalized anticoagulant activity in Figure 7 is calculated as:

(2) (T_sample_ − T_control_)/(T_aptamer_ − T_control_) × 100

For free Apt P studies, buffer was used as control, and T_aptamer_ represents the clotting time of Apt P. For 2HT-BnPn studies, 2HT-Bnnn was used as control, and T_aptamer_ represents the clotting time of 2HT-BnPn. Similarly, for 2HT-BPPB studies, 2HT-BnnB was used as control, and T_aptamer_ represents the clotting time of 2HT-BPPB. Consequently, for normal coagulation, when no aptamer is present in the buffer, the normalized anticoagulant activity is 0%. Similarly, in the absence of any antidote and retriever strands, the normalized anticoagulant activity of the aptamer is 100%.

## 5. Conclusions

In summary, we have successfully demonstrated the use of DNA weave tile and thrombin-binding DNA aptamers to develop a very potent and controllable anticoagulation regime. Molecular dynamics simulations of the weave tiles provided insight into their flexibility and likely interactions with thrombin. We also used more effective thrombin-binding aptamer, Apt P, for anticoagulation studies. Much better anticoagulation was achieved when potent Apt P and Apt B were used on weave tiles. Anticoagulation was significantly improved when multiple copies of these aptamers were displayed on weave tile. The most effective anticoagulants found in this study, 2HT-PPBB and 2HT-BPPB, were more than 16 times better than Apt A, the clinical trial of which was terminated due to low efficacy [9], and almost seven times better than Apt P, currently undergoing clinical trials. We also demonstrated the use of short DNA strands to repeatedly turn the coagulation cascade off and on as desired, thereby providing better control over coagulation. This strategy can be used to target multiple components of the coagulation cascade simultaneously using aptamers, wherein each component could be activated or deactivated independently as desired. Moreover, weave tile or other DNA nanostructures can be used to design distance-dependent multivalent ligands for other targets with therapeutic applications.

## Supplementary Materials

Supplementary materials can be accessed at: http://www.mdpi.com/1420-3049/21/2/202/s1.
