# Supplementary material for: Design of Potent and Controllable Anticoagulants Using DNA Aptamers and Nanostructures
Source: Molecules. 2016 Feb 6;21(2):202. doi: 10.3390/molecules21020202 (PMC6273181; doi:10.3390/molecules21020202)
Supplement: Supplementary file 1 [file molecules-21-00202-s001.pdf]

Supplementary Materials: Design of Potent and Controllable Anticoagulants Using DNA Aptamers and Nanostructures

Abhijit Rangnekar, Jessica A. Nash, Bethany Goodfred, Yaroslava G. Yingling and Thomas H. LaBean

Assembly Constituents and DNA Sequences (5′ to 3′)

Sample	Strands
1HT-BnnA	W1-2_Atop + W1-2_Bbot
1HT-BnAn	W1-2_Atop + W1-1_Bbot
1HT-BnnP	W1-2_Ptop + W1-2_Bbot
1HT-BnPn	W1-2_Ptop + W1-1_Bbot
2HT-AnnB	W2-2_AB + W2-1_noApt
2HT-BnAn	W2-2_Btop + W2-1_Abot
2HT-BAnn	W2-2_Btop + W2-1_Atop
2HT-PnnB	W2-2_PB + W2-1_noApt
2HT-BnPn	W2-2_Btop + W2-1_Pbot
2HT-BPnn	W2-2_Btop + W2-1_Ptop
3HT-BAnn	W3-2_Btop + W 3-1_Atop
3HT-BnAn	W3-2_BA + W 3-1_noApt
3HT-AnnB	W3-2_Atop + W3-1_Bbot
3HT-BPnn	W3-2_Btop + W3-1_Ptop
3HT-BnPn	W3-2_BP + W3-1_noApt
3HT-PnnB	W3-2_Ptop + W3-1_Bbot
4HT-AnnB	W4-2_AB + W4-1_noApt
4HT-BnAn	W4-2_Btop + W4-1_Abot
4HT-BAnn	W4-2_Btop + W4-1_Atop
4HT-PnnB	W4-2_PB + W4-1_noApt
4HT-BnPn	W4-2_Btop + W4-1_Pbot
4HT-BPnn	W4-2_Btop + W4-1_Ptop
2HT-BPPn	W2-2_Btop + W2-1_PP
2HT-PPPB	W2-2_PB + W2-1_PP
2HT-PPBB	W2-2_PB + W2-1_PB
2HT-BPPB	W2-2_BB + W2-1_PP
2HT-Bnnn	W2-2_Btop + W2-1_noApt
2HT-BnnB	W2-2_BB + W2-1_noApt

Strand	Sequence *
Aptamer A	5′ - GGT TGG TGT GGT TGG - 3′
Aptamer B	5′ - AG TCC GTG GTA GGG CAG GTT GGG GTG ACT - 3′
Aptamer P	5′ - CGC CTA GGTTGG GTA GGGTGGTGG CG - 3′
Aptamer B and A connected via 20 base single-stranded linker	5′ - AGT CCG TGG TAG GGC AGG TTG GGG TGA CTT TCT AGT AGT CCT CCG AGT TAT CAG CGT TGG TTG GTG TGG TTG GTT CGC TGA T - 3′
Aptamer B and P connected via 20 base single-stranded linker	5′ - AGT CCG TGG TAG GGC AGG TTG GGG TGA CTT TCT AGT AGT CCT CCG AGT TCG CCT AGG TTG GGT AGG GTG GTG GCG - 3′
W1-2_Atop	5′ - ATC AGC GTT GGT TGG TGT GGT TGG TTC GCT GAT TTC TCG GAG GAC TAC TAG - 3′
W1-2_Bbot	5′ - CT AGT AGT CCT CCG AGT TAG TCC GTG GTA GGG CAG GTT GGG GTG ACT - 3′
W1-1_Bbot	5′ - AGT CCG TGG TAG GGC AGG TTG GGG TGA CTT TCT AGT AGT CCT CCG AG - 3′
W1-2_Ptop	5′ - CG CCT AGG TTG GGT AGG GTG GTG GCG TTC TCG GAG GAC TAC TAG - 3′
W2-1_noApt	5′ - TTC TAG TAG TCC TCC GAG TTT TTA ATC CAT GAT CCT AC TT - 3′
W2-2_AB	5′ - ATC AGC GTT GGT TGG TGT GGT TGG TTC GCT GAT TTC TCG GAG GAC TAC TAG TTT TGT AGG ATC ATG GAT TA TT AG TCC GTG GTA GGG CAG GTT GGG GTG ACT - 3′
W2-2_Btop	5′ - AGT CCG TGG TAG GGC AGG TTG GGG TGA CT TTC TCG GAG GAC TAC TAG TTT TGT AGG ATC ATG GAT TA TT - 3′
W2-1_Abot	5′ - TTC TAG TAG TCC TCC GAG TTT TTA ATC CAT GAT CCT AC TT ATC AGC GTT GGT TGG TGT GGT TGG TTC GCT GAT - 3′
W2-1_Atop	5′ - ATC AGC GTT GGT TGG TGT GGT TGG TTC GCT GAT TTC TAG TAG TCC TCC GAG TTT TTA ATC CAT GAT CCT AC TT - 3′
W2-2_PB	5′ - CG CCT AGG TTG GGT AGG GTG GTG GCG TTC TCG GAG GAC TAC TAG TTT TGT AGG ATC ATG GAT TA TT AG TCC GTG GTA GGG CAG GTT GGG GTG ACT - 3′
W2-1_Pbot	5′ - TTC TAG TAG TCC TCC GAG TTT TTA ATC CAT GAT CCT AC TT CG CCT AGG TTG GGT AGG GTG GTG GCG - 3′
W2-1_Ptop	5′ - CG CCT AGG TTG GGT AGG GTG GTG GCG TTC TAG TAG TCC TCC GAG TTT TTA ATC CAT GAT CCT AC TT - 3′
W2-1_PP	5′ - CG CCT AGG TTG GGT AGG GTG GTG GCG TTC TAG TAG TCC TCC GAG TTT TTA ATC CAT GAT CCT AC TT CG CCT AGG TTG GGT AGG GTG GTG GCG - 3′
W2-1_PB	5′ - CG CCT AGG TTG GGT AGG GTG GTG GCG TTC TAG TAG TCC TCC GAG TTT TTA ATC CAT GAT CCT AC TT AG TCC GTG GTA GGG CAG GTT GGG GTG ACT - 3′
W2-2_BB	5′ - AG TCC GTG GTA GGG CAG GTT GGG GTG ACT TTC TCG GAG GAC TAC TAG TTT TGT AGG ATC ATG GAT TA TT AG TCC GTG GTA GGG CAG GTT GGG GTG ACT - 3′
W3-1_noApt	5′ - TTC TAG TAG TCC TCC GAG TTT TTA ATC CAT GAT CCT ACT TTT GGC TAC AGA CTG CAT T TT - 3′
W3-2_Btop	5′ - AGT CCG TGG TAG GGC AGG TTG GGG TGA CT TTC TCG GAG GAC TAC TAG TTT TGT AGG ATC ATG GAT TAT TTT AAT GCA GTC TGT AGC C TT - 3′
W3-2_Atop	5′ - ATC AGC GTT GGT TGG TGT GGT TGG TTC GCT GAT TTC TCG GAG GAC TAC TAG TTT TGT AGG ATC ATG GAT TAT TTT AAT GCA GTC TGT AGC C TT - 3′
W3-1_Atop	5′ - ATC AGC GTT GGT TGG TGT GGT TGG TTC GCT GAT TTC TAG TAG TCC TCC GAG TTT TTA ATC CAT GAT CCT ACT TTT GGC TAC AGA CTG CAT T TT - 3′
W3-2_BA	5′ - AGT CCG TGG TAG GGC AGG TTG GGG TGA CT TTC TCG GAG GAC TAC TAG TTT TGT AGG ATC ATG GAT TAT TTT AAT GCA GTC TGT AGC C TT ATC AGC GTT GGT TGG TGT GGT TGG TTC GCT GAT - 3′

W3-1_Bbot	5′ - TTC TAG TAG TCC TCC GAG TTT TTA ATC CAT GAT CCT ACT TTT GGC TAC AGA CTG CAT T TT AG TCC GTG GTA GGG CAG GTT GGG GTG ACT - 3′
W3-1_Ptop	5′ - CG CCT AGG TTG GGT AGG GTG GTG GCG TTC TAG TAG TCC TCC GAG TTT TTA ATC CAT GAT CCT ACT TTT GGC TAC AGA CTG CAT T TT - 3′
W3-2_BP	5′ - AGT CCG TGG TAG GGC AGG TTG GGG TGA CT TTC TCG GAG GAC TAC TAG TTT TGT AGG ATC ATG GAT TAT TTT AAT GCA GTC TGT AGC C TT CG CCT AGG TTG GGT AGG GTG GTG GCG - 3′
W3-2_Ptop	5′ - CG CCT AGG TTG GGT AGG GTG GTG GCG TTC TCG GAG GAC TAC TAG TTT TGT AGG ATC ATG GAT TAT TTT AAT GCA GTC TGT AGC C TT - 3′
W4-1_noApt	5′ - TTC TAG TAG TCC TCC GAG TTT TTA ATC CAT GAT CCT ACT TTT GGC TAC AGA CTG CAT TTT TTA GAT CCT ATG CGT TCG TT - 3′
W4-2_AB	5′ - ATC AGC GTT GGT TGG TGT GGT TGG TTC GCT GAT TTC TCG GAG GAC TAC TAG TTT TGT AGG ATC ATG GAT TAT TTT AAT GCA GTC TGT AGC CTT TTC GAA CGC ATA GGA TCT TTA GTC CGT GGT AGG GCA GGT TGG GGT GAC T - 3′
W4-2_Btop	5′ - AGT CCG TGG TAG GGC AGG TTG GGG TGA CTT TCT CGG AGG ACT ACT AGT TTT GTA GGA TCA TGG ATT ATT TTA ATG CAG TCT GTA GCC TTT TCG AAC GCA TAG GAT CTT T - 3′
W4-1_Abot	5′ - TTC TAG TAG TCC TCC GAG TTT TTA ATC CAT GAT CCT ACT TTT GGC TAC AGA CTG CAT TTT TTA GAT CCT ATG CGT TCG TTA TCA GCG TTG GTT GGT GTG GTT GGT TCG CTG AT - 3′
W4-1_Atop	5′ - ATC AGC GTT GGT TGG TGT GGT TGG TTC GCT GAT TTC TAG TAG TCC TCC GAG TTT TTA ATC CAT GAT CCT ACT TTT GGC TAC AGA CTG CAT TTT TTA GAT CCT ATG CGT TCG TT - 3′
W4-2_PB	5′ - CG CCT AGG TTG GGT AGG GTG GTG GCG TTC TCG GAG GAC TAC TAG TTT TGT AGG ATC ATG GAT TAT TTT AAT GCA GTC TGT AGC CTT TTC GAA CGC ATA GGA TCT TTA GTC CGT GGT AGG GCA GGT TGG GGT GAC T - 3′
W4-1_Pbot	5′ - TTC TAG TAG TCC TCC GAG TTT TTA ATC CAT GAT CCT ACT TTT GGC TAC AGA CTG CAT TTT TTA GAT CCT ATG CGT TCG TT CG CCT AGG TTG GGT AGG GTG GTG GCG - 3′
W4-1_Ptop	5′ - CG CCT AGG TTG GGT AGG GTG GTG GCG TTC TAG TAG TCC TCC GAG TTT TTA ATC CAT GAT CCT ACT TTT GGC TAC AGA CTG CAT TTT TTA GAT CCT ATG CGT TCG TT - 3′
Antidote	5′ - CGC ATA GCA T CGC CAC CAC CCT ACC CAA CCT AGG CG - 3′
Retriever I	5′ - ACG CTA TCG ACG CCT AGG TTG GGT AGG GTG GTG GCG ATG CTA TGC G - 3′
Retriever II	5′ - CGC ATA GCA TCG CCA CCA CCC TAC CCA ACC TAG GCG TCG ATA GCG T - 3′

*: Aptamer A with the 7 bp stem is shown in green, Aptamer B is shown in blue, and Aptamer P is shown in red.

Non-Denaturing Polyacrylamide Gel Electrophoresis

Gels contained desired amount of acrylamide (19:1, acrylamide:bisacrylamide) in a buffer consisting of 40 mM Tris acetate (pH 8.0), 2 mM EDTA, and 12.5 mM magnesium acetate (TAE/Mg^{++}). The annealed sample was mixed in equal volume with tracking dye containing TAE/Mg^{++}, 50% glycerol, and 0.2% each of Bromophenol Blue and Xylene Cyanol FF. Gels were run on a Hoefer (Holliston, MA, USA). SE-600 electrophoresis unit at 10 V/cm and cooled via circulating water bath at 4 °C. The gels were then stained with ethidium bromide and imaged using an AlphaImager® (ProteinSimple, Santa Clara, CA, USA).

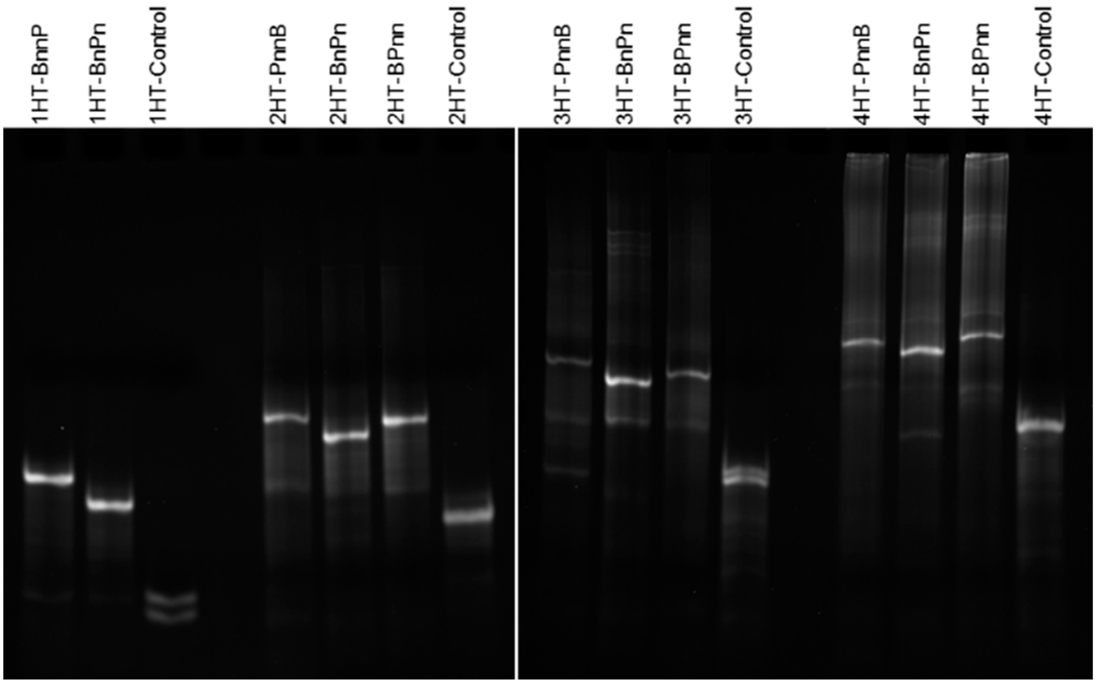

Figure S1. 6% non-denaturing polyacrylamide gel electrophoresis images for characterizing the weave tiles with Apt B and Apt P. The control lane contains two non-complementary strands of same length as the participating strands of the corresponding weave tile. The two non-complementary strands have one aptamer each on them. The slower migration of the most prominent band in the weave tile lane indicates the formation of the weave tile. The gel electrophoresis characterization of the weave tiles with Apt B and Apt A has been shown previously [1].The anticoagulant activity of these constructs is shown in Figure 5 of the main text.

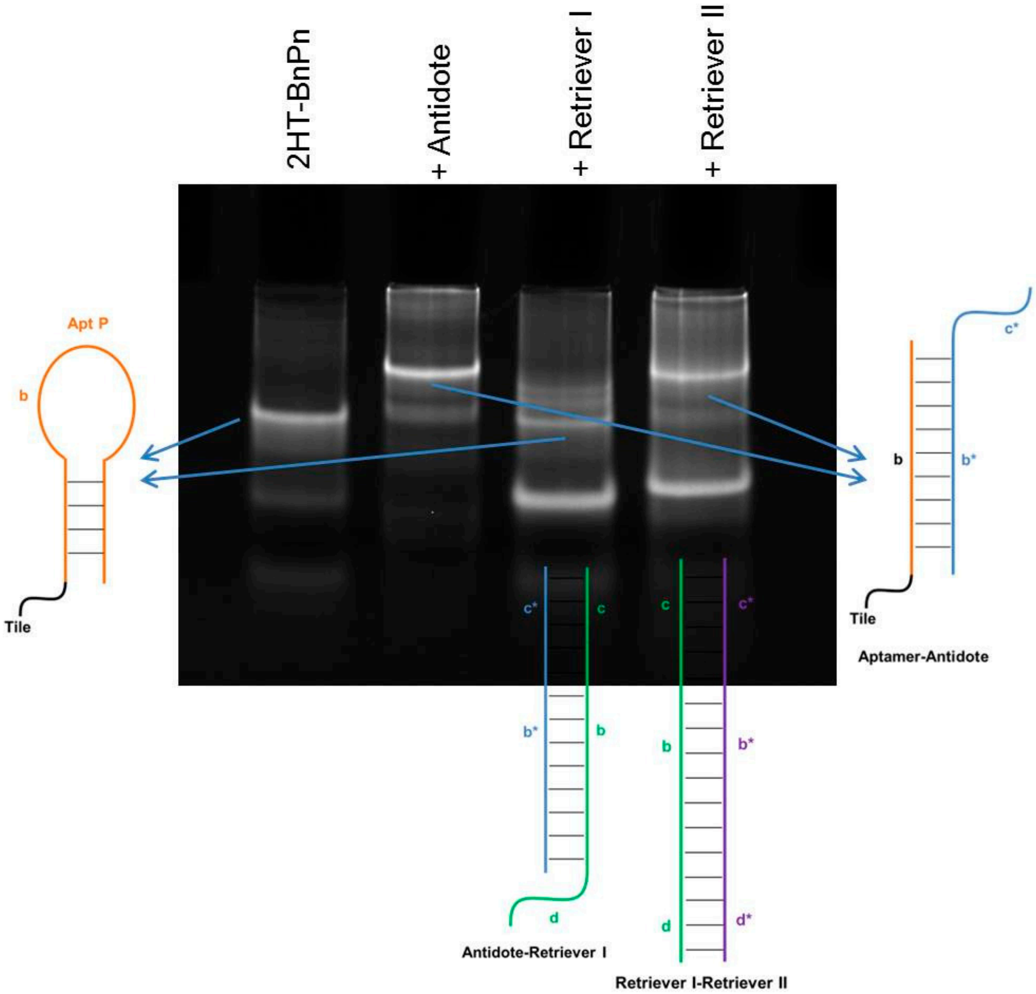

Figure S2. 10% non-denaturing polyacrylamide gel electrophoresis images showing migration of the weave tile 2HT-BnPn (lane 1). Antidote binding to Apt P in 2HT-BnPn resulted in slower migration (lane 2). Addition of Retriever I removed the antidote from the complex (lane 3), whereas, addition of Retriever II removed Retriever I, thus freeing up the antidote, which then bound to 2HT-BnPn again (lane 4). Molar ratio was as follows: 2HT-BnPn: Antidote: Retriever I: Retriever II::1:2:2:2. This strategy is schematically illustrated in Figure 6 of the main text, and the results of clotting assay for the same are shown in Figure 7 of the main text.

Table S1. Clotting time (in seconds) for the data shown in Figure 3 of the main text.

Sample	Trial I	Trial II	Trial III	Trial IV	Trial V	Trial VI	Average	SEM
Buffer Control	19.1	22.6	24.8	18.4	21.1	25	21.83	1.14
Apt B	39.8	39.9	39.3	44.5	43.6	41	41.35	0.89
Apt A	56.4	56.9	56.6	58.7	59.6	62.9	58.52	1.02
Apt P	107.2	110.7	108.1	110.9	114.8	117.7	111.57	1.64
B~A	97	97.3	94.1	98.4	100.2	93.1	96.68	1.08
B~P	189.3	193.1	189.6	172.1	178.5	179.1	183.62	3.35
		Day 1			Day 2			

Table S2. Clotting time (in seconds) for the data shown in Figure 5 of the main text.

Sample	Trial 1	Trial 2	Trial 3	Average (Day 1)	Trial 1	Trial 2	Trial 3	Average (Day 2)	Trial 1	Trial 2	Trial 3	Average (Day 3)
Buffer Control	27.5	26	26.7	26.73	30.4	28.9	28.5	29.27	47.7	46.6	46.2	46.83
Apt B	44.4	37.8	41.4	41.20	40	40	37.9	39.30				
Apt A	62.5	59.9	63.9	62.10	61.1	58.2	53.9	57.73				
Apt P	104.4	104.7	100	103.03	103	101.9	104.3	103.07				
1HT-BnnA	107.5	101	96	101.50					162.3	156.7	160.6	159.87
1HT-BnAn	111.8	107.3	104.4	107.83	114.7	110.9	106.6	110.73				
1HT-BnnP	165.2	164.8	153.3	161.10	167.9	177.9	146.9	164.23				
1HT-BnPn	232.3	212	216.3	220.20	218.5	236.3	231.1	228.63				
2HT-AnnB	113.5	111.9	106.9	110.77	119.7	112.4	109.8	113.97				
2HT-BnAn	115.7	169.7	113.7	133.03	129.8	114.3	104.8	116.30				
2HT-BAnn	115.5	110	111.8	112.43	128.5	118.2	111.5	119.40				
2HT-PnnB	126.6	136.3	101	121.30	143.2	129.8	126.9	133.30				
2HT-BnPn	277	249.7	285.9	270.87	310.6	279.1	366.2	318.63				
2HT-BPnn	223.9	211.7	262.7	232.77	204	212.9	222.6	213.17				
3HT-AnnB	125.5	102.4	103	110.30	145.7	128.3	135.4	136.47				
3HT-BnAn	139.3	125.3	128.7	131.10	153.5	141.5	138.7	144.57				
3HT-BAnn	137.5	129.2	115.6	127.43	152.4	143.9	139	145.10				
3HT-PnnB	243.8	235.9	258.1	245.93	275.1	277.7	363.9	305.57				
3HT-BnPn	270.6	281.3	248	266.63	289.7	274.6	316.4	293.57				
3HT-BPnn	225.4	232.1	206.2	221.23	344.8	251.4	271.7	289.30				
4HT-AnnB	109.6	110.2	111.8	110.53	151.6	150.8	145.4	149.27				
4HT-BnAn	161.2	160.5	166.2	162.63	170.1	161.4	157	162.83				
4HT-BAnn	163.3	163	148.1	158.13	189.9	204.8	188.7	194.47				
4HT-PnnB	351.1	303.6	305	319.90	334.1	337.1	382.2	351.13				
4HT-BnPn	323.5	270.4	301.2	298.37	384.3	369.2	300.1	351.20				
4HT-BPnn	321.2	311.7	316.5	316.47	356.5	336	393	361.83				
Buffer Control (For the following samples)	22.1	23.8	23.8	23.23	23.9	24	25.7	24.53				
2HT-BPPn	318.7	333.3	339.6	330.53	315.4	330.3	329.9	325.20				
2HT-PPPB	376	352	349.8	359.27	348.5	348.5	406.9	367.97				
2HT-PPBB	415.9	422.2	399.8	412.63	420.9	425.1	404	416.67				
2HT-BPPB	397.5	436.8	471.4	435.23	402.3	405.6	411.6	406.50				
			Day 1				Day 2				Day 3	

Table S3. Relative Anticoagulant Activity (RAA) of the samples from Table S2 and as shown in Figure 5 of the main text. The relative anticoagulant activity is calculated as: $(T_{sample} - T_{control})/T_{control}$. Here, T is the clotting time in seconds.

Sample	RAA (Day 1)	RAA (Day 2)	RAA (Day 3)	Average RAA	SEM
Buffer Control	0	0	0		
Apt B	0.541147	0.342825		0.441986	0.099161
Apt A	1.322943	0.972665		1.147804	0.175139
Apt P	2.854115	2.52164		2.687877	0.166237
1HT-BnnA	2.796758		2.413523	2.605141	0.191617
1HT-BnAn	3.033666	2.783599		2.908632	0.125033
1HT-BnnP	5.026185	4.611617		4.818901	0.207284
1HT-BnPn	7.236908	6.812073		7.02449	0.212417
2HT-AnnB	3.143392	2.894077		3.018734	0.124657
2HT-BnAn	3.976309	2.973804		3.475057	0.501253
2HT-BAnn	3.205736	3.079727		3.142731	0.063005
2HT-PnnB	3.537406	3.55467		3.546038	0.008632
2HT-BnPn	9.13217	9.887244		9.509707	0.377537
2HT-BPnn	7.706983	6.283599		6.995291	0.711692
3HT-AnnB	3.125935	3.66287		3.394403	0.268467
3HT-BnAn	3.90399	3.939636		3.921813	0.017823
3HT-BAnn	3.766833	3.957859		3.862346	0.095513
3HT-PnnB	8.199501	9.440774		8.820138	0.620637
3HT-BnPn	8.973815	9.030752		9.002284	0.028468
3HT-BPnn	7.275561	8.884966		8.080263	0.804702
4HT-AnnB	3.134663	4.100228		3.617446	0.482782
4HT-BnAn	5.083541	4.563781		4.823661	0.25988
4HT-BAnn	4.915212	5.644647		5.279929	0.364717
4HT-PnnB	10.96633	10.99772		10.98203	0.015694
4HT-BnPn	10.16085	11		10.58042	0.419576
4HT-BPnn	10.83791	11.36333		11.10062	0.26271
Buffer Control (For the following samples)	0	0			
2HT-BPPn	13.22669	12.25543		12.74106	0.485626
2HT-PPPB	14.46341	13.99864		14.23103	0.232387
2HT-PPBB	16.7604	15.9837		16.37205	0.388353
2HT-BPPB	17.73314	15.56929		16.65122	1.081924

Table S4. Clotting time (in seconds) for the data shown in Figure 7 of the main text. AD: antidote; R-I: Retriever I; R-II: Retriever II.

Sample	Trial I	Trial II	Trial III	Average (Day 1)	Trial IV	Trial V	Trial VI	Average (Day 2)
Buffer Control	23.6	23.8	23.5	23.63	23.6	23.9	24	23.83
Apt P	91.7	95	96.8	94.50	96.2	95.8	95.3	95.77
Apt P + AD	26.9	26.8	26.8	26.83	27.6	27.8	27.4	27.60
Apt P + AD + R-I	78.9	70.4	76.5	75.27	87.7	83.9	87.8	86.47
Apt P + AD + R-I + R-II	31.2	31.9	31.3	31.47	32	32.4	32.9	32.43
2HT-Bnnn	42.4	42.3	42.6	42.43	46	46.6	46.7	46.43
2HT-BnPn	297.8	293.3	304.5	298.53	297.8	309.1	310.2	305.70
2HT-BnPn + AD	50.1	51	51.1	50.73	52.8	53.4	55.6	53.93
2HT-BnPn + AD + R-I	241.6	246.5	208.7	232.27	242.6	266.2	275.5	261.43
2HT-BnPn + AD + R-I + R-II	62.4	63.5	62.9	62.93	64.2	64.9	66.2	65.10
2HT-BnnB	61.6	63.5	63.4	62.83	66.2	68.4	68.8	67.80
2HT-BPPB	685.2	699.7	757.9	714.27	648.9	711.5	735.9	698.77
2HT-BPPB + AD	89.5	84.9	86.4	86.93	85.8	87.5	89.2	87.50
2HT-BPPB + AD + R-I	512.1	539.3	548.8	533.40	599.3	628.4	620.1	615.93
2HT-BPPB + AD + R-I + R-II	90.2	92.5	94.2	92.30	109.9	101.7	100.7	104.10
			Day 1				Day 2	

Table S5. Anticoagulant Activity (AA, in percentage) of the samples from Table S4 and as shown in Figure 7 of the main text. The anticoagulant activity is calculated as: $(T_{sample} - T_{control})/(T_{aptamer} - T_{control}) \times 100$. For free Apt P studies, buffer was used as control, and $T_{aptamer}$ represents the clotting time of Apt P. For 2HT-BnPn studies, 2HT-Bnnn was used as control, and $T_{aptamer}$ represents the clotting time of 2HT-BnPn. Similarly, for 2HT-BPPB studies, 2HT-BnnB was used as control, and $T_{aptamer}$ represents the clotting time of 2HT-BPPB.

Sample	AA (Trial I)	AA (Trial II)	AA (Trial III)	AA (Trial IV)	AA (Trial V)	AA (Trial VI)	Average AA	SEM
Buffer Control	−0.05	0.24	-0.19	−0.32	0.09	0.23	0.00	0.09
Apt P	96.05	100.71	103.25	100.60	100.05	99.35	100.00	0.96
Apt P + AD	4.61	4.47	4.47	5.24	5.51	4.96	4.88	0.18
Apt P + AD + R-I	77.99	65.99	74.60	88.79	83.50	88.92	79.97	3.64
Apt P + AD + R-I + R-II	10.68	11.67	10.82	11.35	11.91	12.60	11.50	0.29
2HT-Bnnn	−0.01	-0.05	0.07	−0.17	0.06	0.10	0.00	0.04
2HT-BnPn	99.71	97.96	102.33	96.95	101.31	101.74	100.00	0.89
2HT-BnPn + AD	2.99	3.35	3.38	2.46	2.69	3.54	3.07	0.18
2HT-BnPn + AD + R-I	77.77	79.68	64.92	75.66	84.76	88.35	78.53	3.32
2HT-BnPn + AD + R-I + R-II	7.80	8.23	7.99	6.85	7.12	7.62	7.60	0.21
2HT-BnnB	−0.19	0.10	0.09	−0.25	0.10	0.16	0.00	0.07
2HT-BPPB	95.54	97.76	106.70	92.10	102.02	105.89	100.00	2.39
2HT-BPPB + AD	4.09	3.39	3.62	2.85	3.12	3.39	3.41	0.17
2HT-BPPB + AD + R-I	68.97	73.14	74.60	84.24	88.85	87.53	79.55	3.41
2HT-BPPB + AD + R-I + R-II	4.20	4.55	4.82	6.67	5.37	5.21	5.14	0.35
		Day 1			Day 2			

References

1. Rangnekar, A.; Zhang, A.M.; Li, S.S.; Bompiani, K.M.; Hansen, M.N.; Gothelf, K.V.; Sullenger, B.A.; LaBean, T.H. Increased anticoagulant activity of thrombin-binding DNA aptamers by nanoscale organization on DNA nanostructures. *Nanomedicine* **2012**, *8*, 673–681.
